# Transient Expression of Zika NS2B Protein Antigen in *Nicotiana benthamiana* and Use for Arboviruses Diagnosis

**DOI:** 10.1021/acsomega.4c08998

**Published:** 2025-01-08

**Authors:** Mario A. M. Herazo, Daylana R. S. Dantas, Bruno B. Silva, Helen P. S. Costa, Eduarda N. F. N. Santos, Luiz F. W. G. Moura, João X. S. Neto, Maurício F. Van Tilburg, Eridan O. P. T. Florean, Arlindo A. Moura, Maria I. F. Guedes

**Affiliations:** † Laboratory of Biotechnology and Molecular Biology, Health Sciences Center, State University of Ceara, Fortaleza 60714-903, Brazil; ‡ Federal University of Ceara, Fortaleza 60355-636, Brazil; § Federal Rural University of the Semi-Arid, Mossoro 59625-900, Brazil

## Abstract

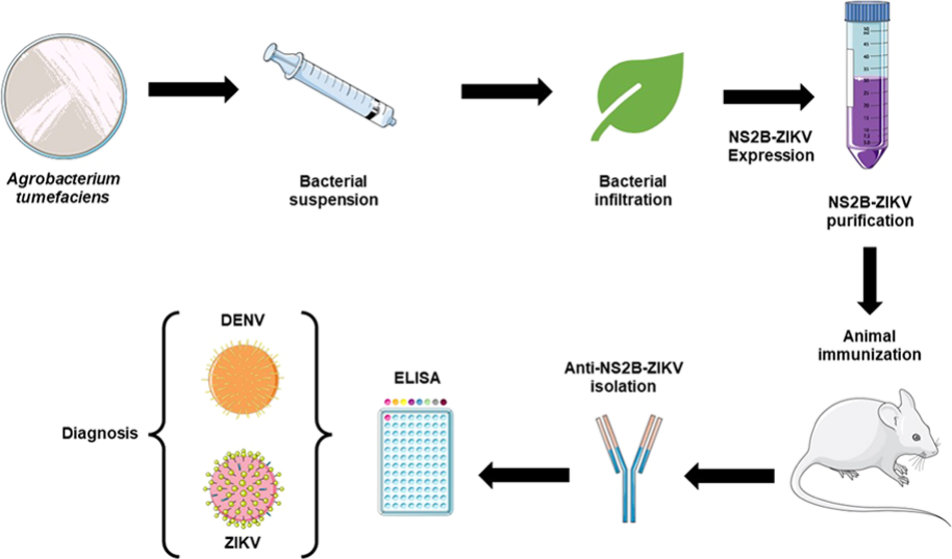

Zika (ZIKV) and Dengue (DENV) viruses are clinically significant due to their severe neurological and hemorrhagic complications. Rapid diagnostics often rely on nonstructural proteins to generate specific antibodies. This study aimed to produce IgG antibodies from the recombinant ZIKV protein and plant-expressed NS2B protein for arbovirus detection in serum and urine samples. The NS2B protein was expressed in *Nicotiana benthamiana* and purified chromatographically. Validation of recombinant NS2B as an antigen in indirect immunoassays demonstrated 95% sensitivity and 100% specificity in IgM/IgG ELISA tests, enabling effective detection of ZIKV and DENV. Notably, r-ZIKV-NS2B IgG identified positive ZIKV and DENV cases in urine but failed to detect negatives, suggesting limitations in specificity for urine diagnostics. Using urine as a diagnostic medium offers a less invasive and more practical approach, broadening the test applicability. This study utilized patient-derived positive urine samples and healthy samples spiked with an exogenous virus. Findings highlight the potential of the ZIKV-NS2B protein as a robust antigen for arbovirus diagnosis and demonstrate the viability of plant-based systems for antigen production, advancing diagnostics for neglected tropical diseases.

## 1 Introduction

Flaviviruses are a group of viral agents that together comprise the Flaviviridae family. These viruses are characterized by having a viral envelope and genetic material composed of a single strand of positive RNA.^1−3^ Within the flavivirus family, arboviruses (arthropod-borne viruses) deserve special attention. This group is made up of viruses that have arthropods as their transmission vector and development cycle. Among the various arboviruses, some emerge as emerging and cause neglected diseases, such as Dengue, Zika, Chikungunya, and Racio, among other virus.^4−7^

In this context, Zika is an emerging infectious disease caused by the Zika virus (ZIKV), an RNA virus transmitted mainly by infected *Aedes aegypti* mosquitoes. It can also be sexually transmitted or by receiving blood products.^8−10^ Another arbovirus of clinical relevance is the Dengue virus. This infectious agent also has a *A. aegypti* as a transmission vector. However, similar to the Zika virus, some studies have also reported the presence of the Dengue virus in seminal fluid samples, being correlated with possible transmission through sexual fluids.^11−13^

The Dengue and Zika viruses are responsible for epidemics that generate major impacts on public health bodies. According to the WHO, there has been an increase of more than 500% in the notification of Dengue cases in the last 20 years, with no prospect of reduction.^14^ It is also estimated that approximately 40% of the world’s population is in areas at high risk of Dengue transmission.^15^ Zika epidemics of 2015–2016 led to the spread of the disease in the Americas and tropical countries. Specifically in Brazil, the infection has been associated with neurological complications such as microcephaly in newborns and Guillain–Barré syndrome in adults.^16−20^

In addition to the numerous existing cases, a risk factor for the increase in the number of Dengue and Zika cases is related to climate change. This is due to the fact that there is an increase in global temperatures and changes in rainfall rates and distribution, with the potential to provide a more favorable environment for the development of the *A. aegypti* vector of both viruses.^21−24^ In this context, it is clear that the Dengue and Zika epidemics are of great concern to public authorities, and their clinical diagnosis is of great importance.

One of the tests that can be used to determine Dengue and Zika infections is a test that detects specific antibodies or antigens. Based on this, the nonstructural proteins (NS1, NS2A, NS2B, NS3, NS4A, NS4B, and NS5) present in the Zika virus have shown great potential to be used as specific antigens to be detected in diagnostic kits.^25−27^ Continuing on this topic, we have the NS2B protein, which is a cofactor for the NS3 serine protease and forms the NS2B/NS3 complex, which plays an essential role in the hydrolysis and maturation of the viral polyprotein. These proteins are found in the intracellular compartment of the host cell and are primarily associated with the membranes of the endoplasmic reticulum during ZIKV replication. Their function is essential for the survival and spread of the virus, making them attractive targets for the development of antiviral therapies and diagnostics.^28−31^ It presents a 20 residue peptide epitope (DITWEKDAEVTGNSPRLDVA) specific for ZIKV, which has supported a sensitive and specific diagnosis of the disease in infected patients.^32^

The production of recombinant antigens for serological testing has enabled the facilitation and improvement of diagnostic systems that are in use today. Plants have been used to produce proteins that are used as diagnostic reagents, vaccines, and drugs.^33^ This process, also referred to as molecular farming, has shown the potential of this platform to increase antigen production (scalable production) and reduce costs.^34^

Our research groups have proposed the use of the plant platform as a means of production for viral antigens that can be used for the development of diagnostic kits, using the protein NS1 from Dengue virus and NS2B from Zika virus.^33,35^ Based on the above, this research aims to carry out the transient expression of the Zika virus NS2B protein on a vegetable platform, aiming at the production of mouse polyclonal antibodies for the detection of arbovirus viruses (Dengue and Zika) in urine and blood samples.

## 2 Results and Discussion

### 2.1 r-ZIKV-NS2B Protein Expression in *Nicotiana benthamiana*

**Figure 1:**
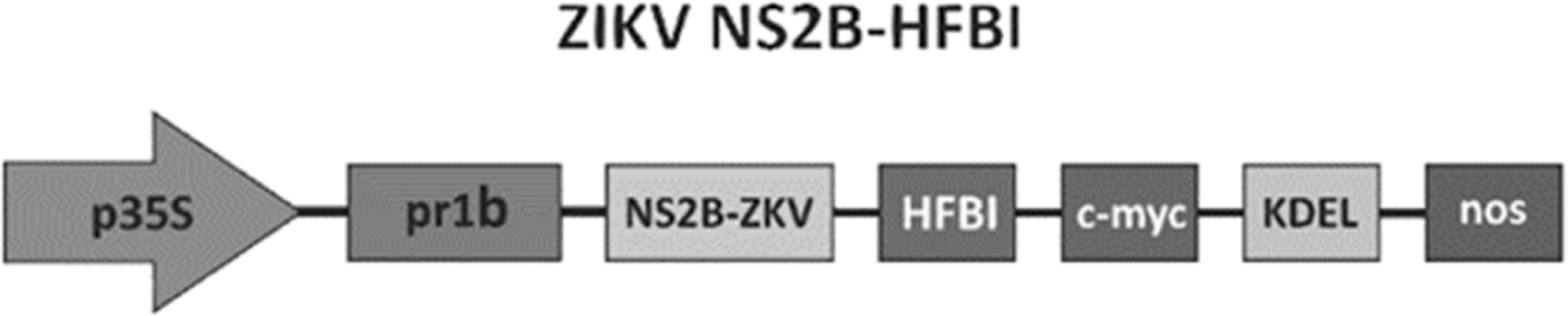
Schematic representation for the expression of recombinant ZIKV-NS2B. Schematic representation of the ZIKV-NS2B-HFI construct. p35S, double enhanced 35S promoter from Cauliflower Mosaic Virus 35S gene; Pr1b, tobacco pathogenesis-related 1b protein secretory signal peptide; NS2B-ZIKV, optimized sequence NS2B of ZIKV; HFBI, hydrophobin I; c-Myc, detection/purification tag; KDEL, endoplasmic reticulum retrieval tetrapeptide; and nos, nopaline synthase transcription terminator.

In this research, codon optimization was used to express a larger amount of the *r-NS2B-HFBI* protein in *N. benthamiana*.^36−38^ Of the 127 codons that make up ZIKV-NS2B, 43 codons remained unchanged, 71 codons were modified by one nucleotide, eight codons were modified by two nucleotides, and five codons were completely modified (Figure 1). These types of modifications are necessary in some cases, such as for hemagglutinin A (HA) from avian influenza virus H5N1, which was discovered only after codon optimization for its expression in *N. benthamiana*.^39^ To this end, it was used as a source of codon optimization for expression in plants, and the endoplasmic reticulum address ER was maintained to ensure that the synthetic polypeptide would be appropriately expressed.

After optimization, the *r-ZIKV-NS2B* sequence was synthesized and cloned into the pCAMGate-ER-HFBI plasmid for expression as a construct fused to the HFBI tag at the C-terminus. The tetrapeptide KDEL (Lys–Asp–Glu–Leu), also present in this construct, promoted the retention of the recombinant protein in the endoplasmic reticulum of the plant cell. Although the specific effect of the KDEL tag on the degree of accumulation of the r-NS2B-HFBI construct was not examined in this work, there is ample evidence that ER-directed proteins are passively sequestered in protein bodies that confer greater stability to the recombinant proteins.^40,41^ The low protease activity in the endoplasmic reticulum, combined with the presence of chaperones and the machinery for disulfide bond formation, contributes to the stability, folding, and assembly of heterologous proteins.^41−43^

For the expression of r-ZIKV-NS2B, the cultures of *Agrobacterium* with expression vector to the protein was coagroinfiltrated into *N. benthamiana*, along with a construct containing the p19 suppressor for gene silencing from *Cymbidium virus* ringSpot (p19).^44^ This is one of the most common silencing inhibitors used to increase the transcription and protein accumulation in transient processes.^41,45,46^ For GFP protein, it was reported that the expression level increased 50-fold when p19 was present.^47^

**Figure 2:**
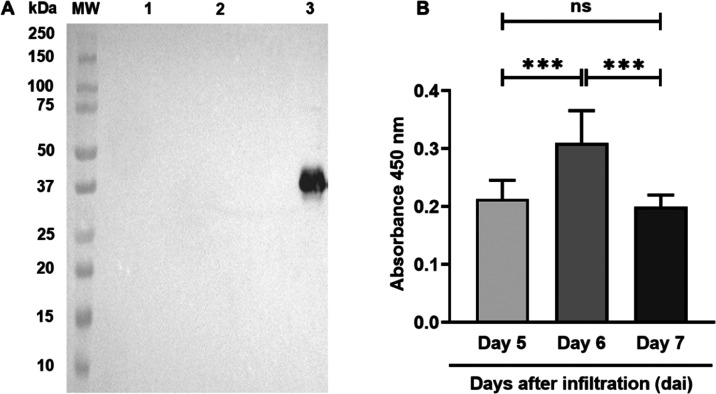
Extraction and detection of r-ZIKV-NS2B expressed in *Nicotiana benthamiana*. (A) Detection of the r-ZIKV-NS2B protein using the anti-c-Myc antibody. Lane 1: extract of noninfiltrated leaves; Lane 2: Extract of p19 infiltrated leaves (control plant); Lane 3: r-NS2B-HFBI infiltrated leaves, where a single 31 kDa band could be detected. (B) Over time accumulation of the r-ZIKV-NS2B construct on infiltrated leaves. A standardized amount of leaf extract containing soluble proteins (40 μg/well) was added to each well. Wells 1 and 2 were used as negative controls to demonstrate that the r-ZIKV-NS2B protein appeared as a result of infiltration. Tukey’s Multiple Comparison Test did not show a difference between the evaluated time points (*p* > 0.05) of viral samples.

After agroinfiltration, the r-ZIKV-NS2B level of protein expression was determined at 5, 6, and 7 dpi empirically. For the detection of r-ZIKV-NS2B, total soluble proteins (TSP) were extracted from infiltrated leaves of *N. benthamiana,* and SDS-PAGE and Western blot were performed for protein detection. After the electrophoresis process, a single-band protein (with approximately 31 kDa) was detected by Western blotting against the c-myc tag in r-ZIKV-NS2B expressed in infiltrated leaves. At the same time, the presence of the r-ZIKV-NS2B protein was not detected in noninfiltrated leaves (negative control) (Figure 2A).

Normally, the recombinant protein accumulates in detectable amounts between 3 and 8 days after infiltration.^48,49^ However, because protein expression was undetectable up to 4 days after infiltration and necrosis of infiltrated leaves began 8 days after infiltration, only r-ZIKV-NS2B expression in the time interval between 5 and 7 days after infiltration was examined in this work. The TSP from leaves at 5–7 days after agroinfiltration were analyzed by ELISA to evaluate the dynamics of the NS2B-HFBI-ER expression and determine the best time for the harvest of the produced proteins.

ELISA assay detection demonstrated that among the days evaluated following infiltration (5, 6, and 7), the period showing the highest detection level was day 6. This is evident from the statistical difference observed between days 6 and 5 and between days 6 and 7, while days 5 and 7 are not statistically different. Thus, the leaves used for the recombinant protein purification process were harvested on day 7 (Figure 2B).

Many factors can influence the level of transient expression and the yield of recombinant proteins after the agroinfiltration process.^50,51^ For example, the best time to harvest infiltrate leaves should be empirically tested to determine the time with the highest level of protein accumulation. Norkunas and colleagues^48^ examined the activity of the enzyme GUS in infiltrated leaves, finding conditions like those in this work. They collected leaves at 0, 2, 4, 6, and 8 dpi. And these authors reported the highest GUS activity between 4 and 6 days after infiltration. However, by the 8th day, the enzyme was likely degraded, as the expression of GUS was no longer detectable by fluorimetry.

### 2.2 r-ZIKV-NS2B Purification

Despite all of these advantages, protein purification is the major bottleneck in the production of a recombinant protein-based product, leading to an overall increase in cost. Therefore, hydrophobin I (HFBI) from *Trichoderma reesei* was expressed as a fusion with proteins of interest to facilitate its recovery from plant leaf extracts, which is done by the Aqueous Two-Phase System (ATPS), recovering the protein efficiently without using chromatographic methods.^34,52,53^

Hydrophobin I fusion is a simple strategy that allows the purification of recombinant proteins from different platforms.^54^ Jacquet et al.^55^ were the first to report the high-level expression of a recombinant viral protein fused with hydrophobin I for the development of a vaccine. According to these authors, plant transient expression of the hemagglutinin ectodomain of influenza A/Texas/05/2009 (H1N1) resulted in 2.5% higher expression when fused to HFBI compared to its nonfused counterpart. These results contrast with those of Phan et al.,^56^ who reported no benefit of fusion to HFBI on the expression of the same domain from influenza A/Hatay/2004 (H5N1) virus in transgenic *N. tabacum*. Although both authors produced similar proteins in related plants, it is difficult to compare their results because the transgenic and transient systems are quite different. If there is an unknown advantage to produce HFBI-fused proteins on the transient expression system, we believe that the r-ZIKV-NS2B construct would have exploited this advantage.

For recombinant protein purification, first, an Aqueous Two-Phase System (ATPS) was optimized for semipurification of the r-ZIKV-NS2B protein using different concentrations of the surfactant Triton X-114 (2, 4, 6 and 8%). At 4 and 8%, the number of native proteins that were copurified with r-NS2B-HFBI was lower than at 2 and 6%. Since the highest concentrations of the surfactant resulted in a lower yield of recovered protein, the 4% concentration was selected as the one with the highest enrichment and the lowest amount of copurified impurities (Figure 2A). Added to this, interestingly, at concentrations of 2 and 6% Triton X-114, protein bands with a mass of approximately 63 kDa were observed. This mass would likely refer to the aggregation of the recombinant protein (approximately 31 kDa) into dimers.

**Figure 3:**
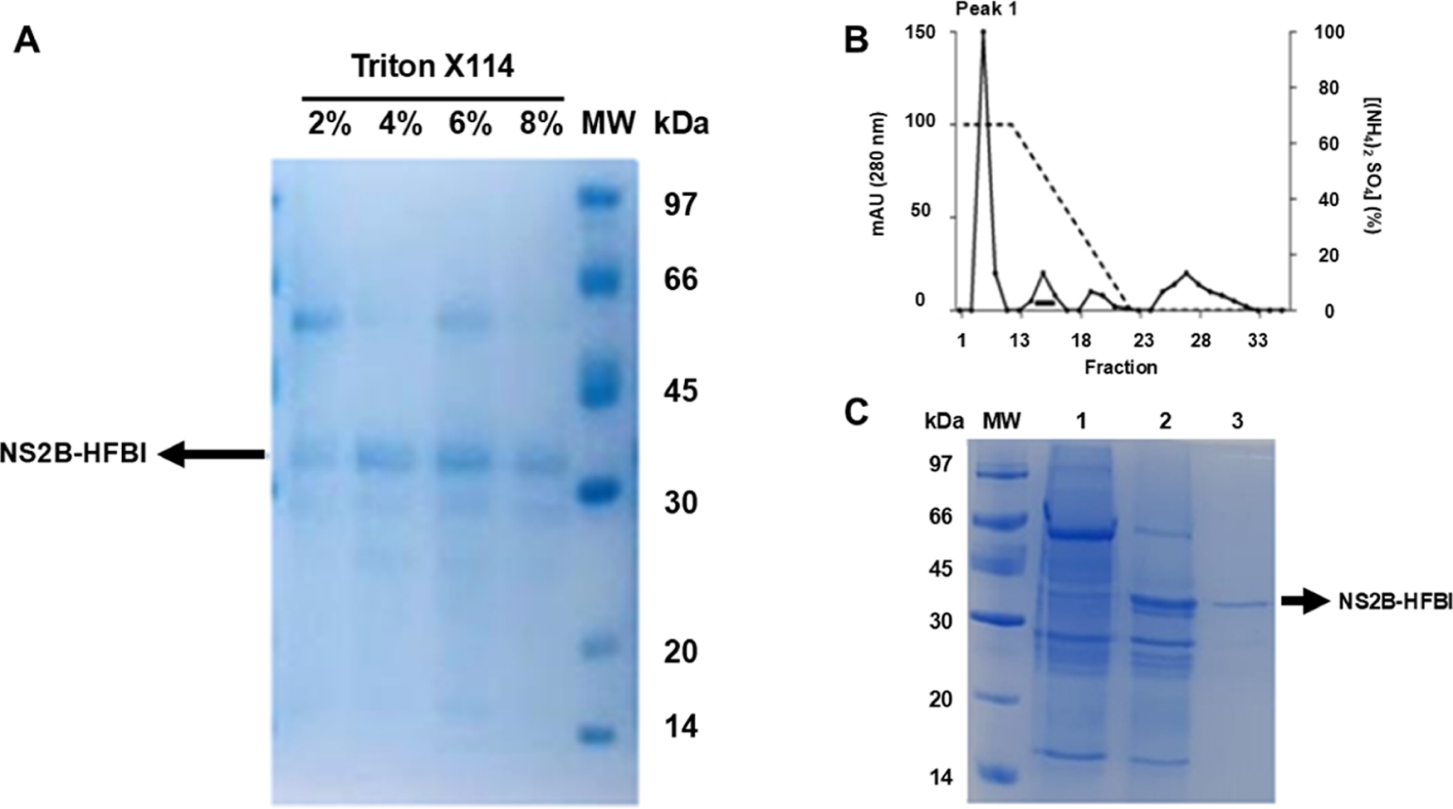
r-ZIKV-NS2B-HFB purification. (A) CBB stained 15% SDS-PAGE gel image showed the ATPS the best concentration of the Triton X-114 surfactant was evaluated (2, 4, 6, and 8%). The 4% concentration showed the highest enrichment and least copurified contaminants. (B) Hydrophobic interaction chromatography, HIC (HiTrap Phenyl HP) from the HFB fraction. HFB fraction (2.45 g) was loaded onto the Phenyl HP column previously equilibrated with 20 mM sodium phosphate buffer (pH 7.4) containing 1 M ammonium sulfate. The r-NS2B-HFB adsorbed peak is identified by the horizontal bar. (C) CBB stained 15% SDS-PAGE gel image from leaf extract of plants infiltrated with r-NS2B-HFB of Zika virus (line 1), r-NS2B-HFB prepurified by ATPS, where enrichments of the protein of interest is also observed (line 2), and r-NS2B-HFB purified by HIC (line 3).

Continuing with the purification process, for this reason, additional steps were required to obtain the purified product. Therefore, the semipurified ATPS fraction was quantified and loaded onto a hydrophobic interaction chromatography (HIC) coupled to the KTATM Start System (GE). The semipurified ATPS fraction (2.45 mg) was loaded onto a phenyl column HIC (HiTrap Phenyl HP, GE), from which four chromatographic peaks were obtained (Figure 3B). The first peak corresponded to the nonretained fraction eluted with 1 M ammonium sulfate solution, whereas the subsequent peaks corresponded to the retained proteins. The r-ZIKV-NS2B protein was detected in the second peak, which eluted along an ammonium sulfate gradient (Figure 3B). The analysis by SDS-PAGE and Western blotting of this fraction revealed a single protein band of 31 kDa. This protein band can be observed in the extract of the infiltrated plant, during the prepurification process, and independently in the eluate peak from the chromatography. These results clearly demonstrate that the purification process was efficient and successfully purified the target protein, resulting in a sample with a high degree of purity (Figure 3C).

Many plants contain some native hydrophobic proteins, so some proteins are copurified by ATPS.^53,57−59^ For this reason, it is necessary to use different concentrations of ammonium sulfate to separate proteins with different levels of hydrophobicity. Interestingly, the use of the hydrophobic tail provided high hydrophobicity for the protein, to the point that the recombinant protein was isolated in peak 1 (highest concentration of ammonium sulfate), and the remaining retained proteins were eluted with much smaller contractions of ammonium sulfate.

**Figure 4:**
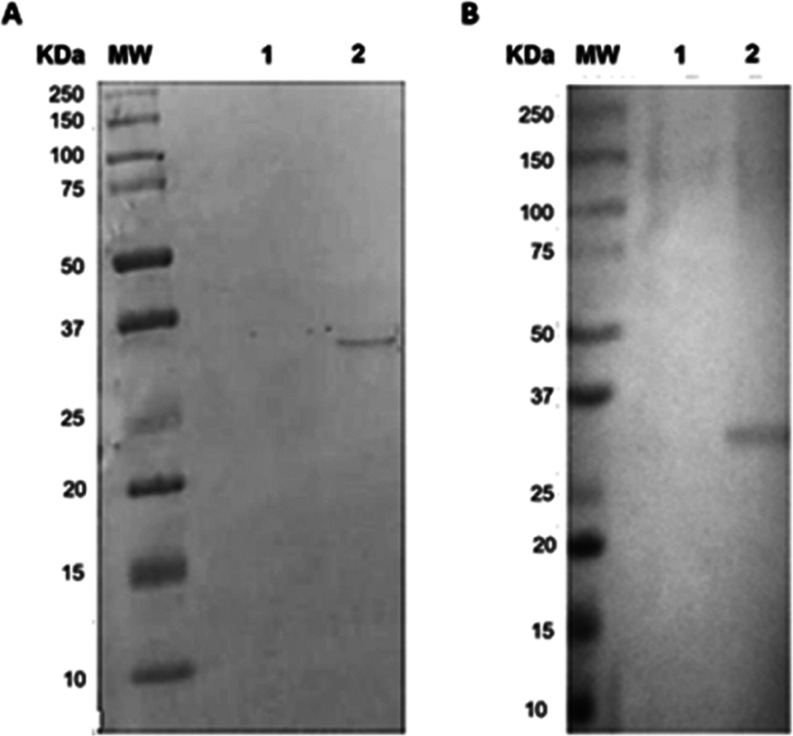
Western blot analysis of r-ZIKV-NS2B produced in *N. benthamiana*. (A) Probed against a commercial polyclonal anti-NS2B antibody (GTX133308, Genetex). (B) Probed against the pooled sera (*n* = 4) of Zika fever patients. MW: molecular weight; Lane 1: Control plants extract; and Lane 2: purified r-ZIKV-NS2B.

Recognition of the purified r-ZIKV-NS2B by a polyclonal antibody targeting a sequence within the central region of the native ZIKV-NS2B protein confirmed that the antigenic nature of the recombinant construct was at least partially retained (Figure 4). This result was verified by a Western blotting assay using pooled sera (*n* = 4) from ZIKV-infected patients, which also detected a single 31 kDa band (Figure 4B). Taken together, these results underscore the potential of the r-ZIKV-NS2B construct for the Zika fever diagnosis. Those results corroborate with the ones related by Ravichandran et al.,^60^ which demonstrated that NS2B is highly reactive for acute and convalescent ZIKV-positive serum and very low reactivity for convalescent DENV-positive serum.

### 2.3 Enzyme-Linked Immunosorbent Assay (ELISA)

Other recombinant proteins expressed in *N. benthamiana* have already been used for the diagnosis of viral diseases.^33,35,61−64^ Marques et al.^64^ reported that cloning the Dengue virus NS1 protein into the same plasmid vector allowed the expression and detection of an HFBI-fused NS1 protein in *N. benthamiana*.^33^ However, the protein produced was insoluble under the conditions tested, hindering its use in the development of diagnostic tests.

**Figure 5:**
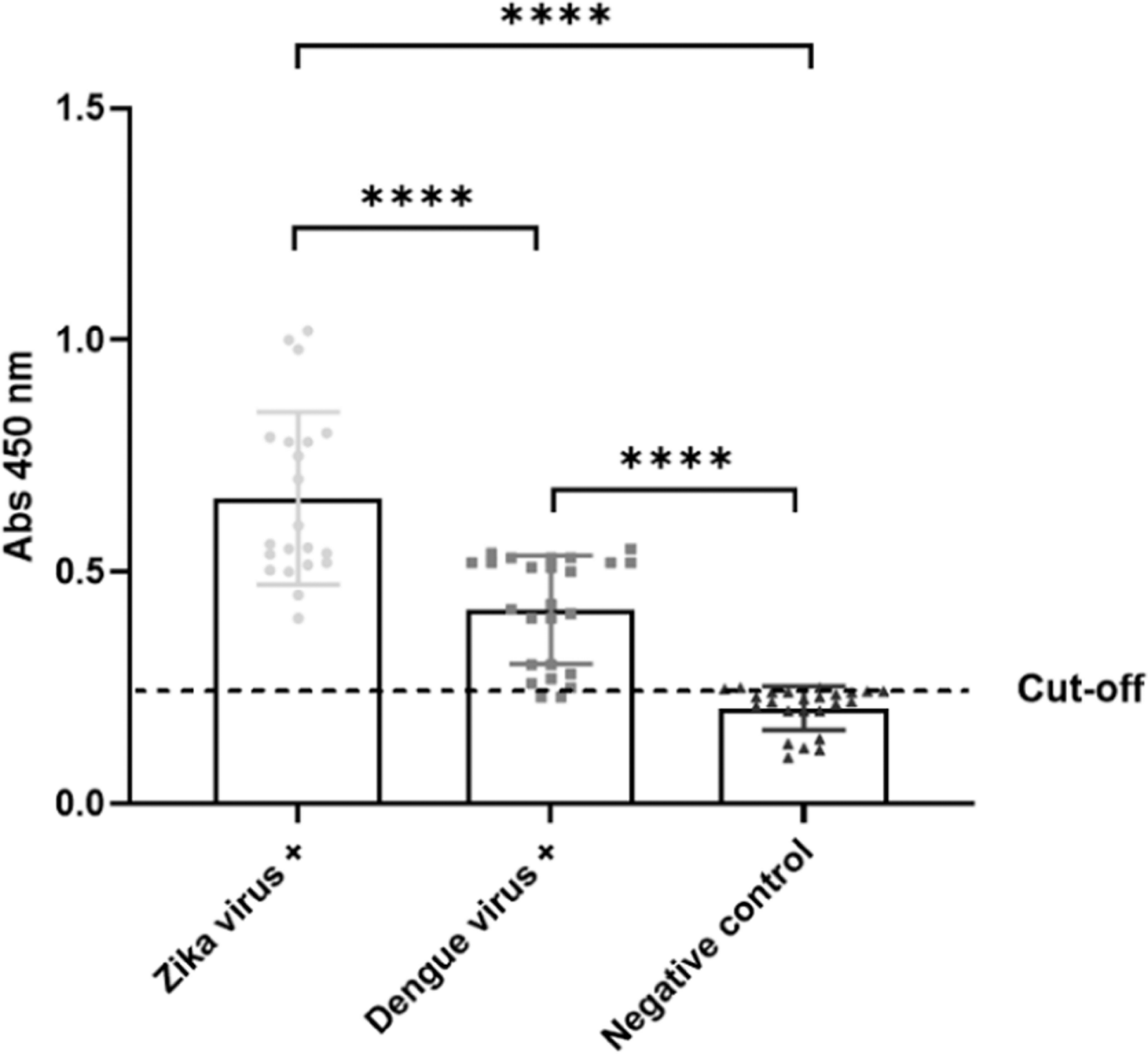
Reactivity of IgM antibody with r-NS2B-HFBI. Sera of patients with Zika fever (*n* = 20) or Dengue fever (*n* = 25) were tested by ELISA. Sera from healthy children with no previous history of infection by Zika or Dengue virus were used as control (*n* = 13). The Tukey test was used as a post-test. Different letters represent statistical differences between groups (*p* < 0.05).

The usefulness of the recombinant r-ZIKV-NS2B as a diagnostic antigen was demonstrated in an IgM ELISA that distinguished healthy sera from those of ZIKV-infected patients, confirmed by RT-qPCR. The developed assay showed a sensitivity of 95% (one false-negative result in 20 positive sera) and a specificity of 100% (no false-positive result in 13 sera) in the tested samples, confirming the use of the transient expression of the r-ZIKV-NS2B construct in plants for the diagnosis of ZIKV infection. However, the same antigen showed some degree of cross-reactivity with sera from DENV-infected patients, although the mean absorbance of each group was statistically different (Figure 5).

According to ref (65), IgM levels are variable during Zika virus infection. In general, titers of this immunoglobulin rise to detectable levels around day 4 after the onset of symptoms and remain detectable until 12 or more weeks after the initial infection. Therefore, detection of IgM is a valuable tool for diagnosing symptomatic or asymptomatic patients in the early stages of infection, especially when molecular techniques fail to detect ZIKV RNA due to low viremia.^66^

One of the strengths of this work was the evaluation of the r-ZIKV-NS2B construct against a library of sera from individual groups. The group of Zika fever-positive patients included only patients diagnosed by RT-qPCR, the gold standard test for Zika, as well as many other viral diseases. The Dengue fever-positive group was composed of sera from individuals diagnosed several years before the first report of Zika fever in Brazil, virtually eliminating the risk of cross-reactivity due to undiagnosed infection with ZIKV. The control group included sera from children who had no history of viral infection. Although these children may have been asymptomatic for previous infections, the low absorption in the tests using the r-ZIKV-NS2B construct underscores their usefulness as a healthy control group.

**Figure 6:**
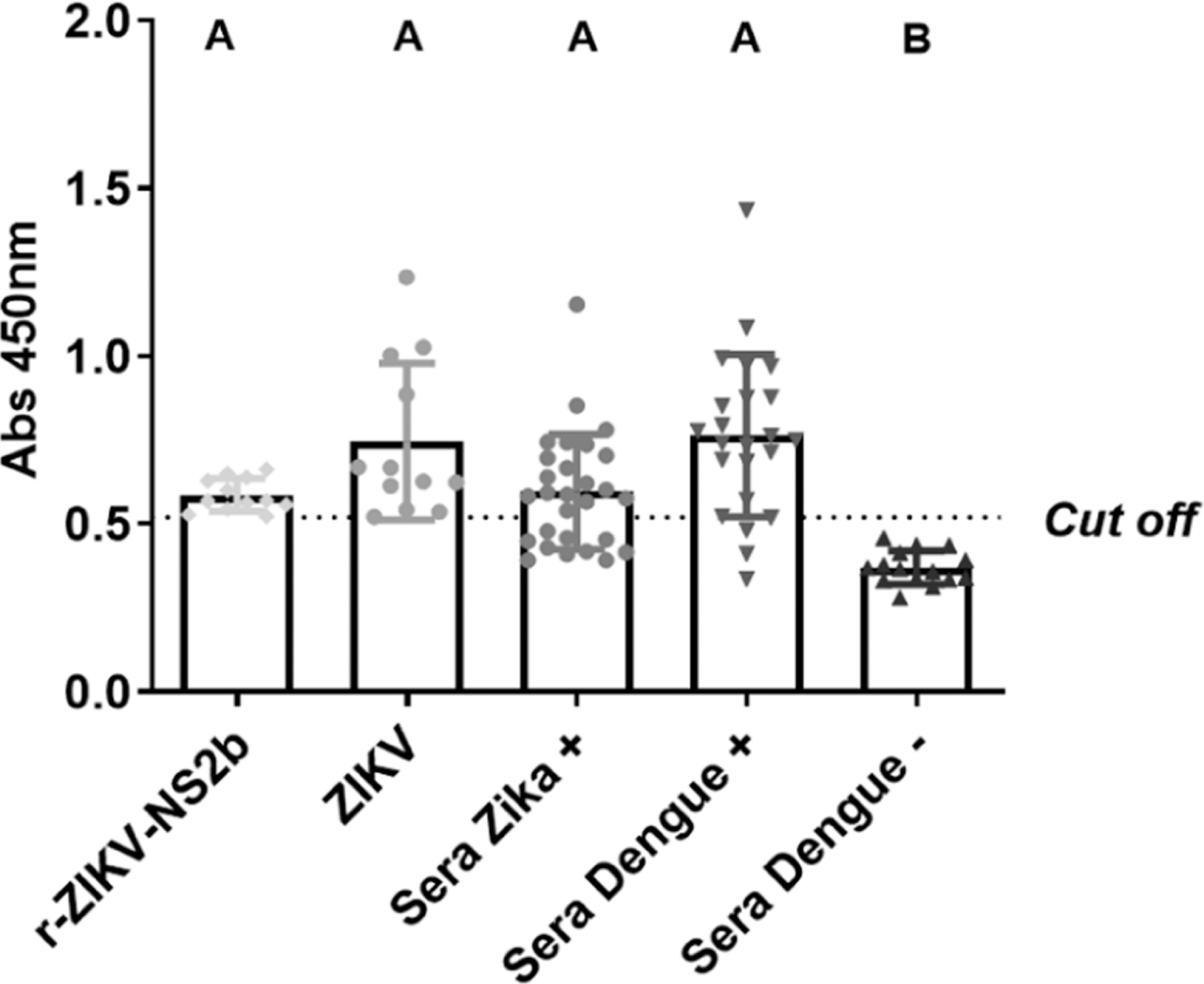
Reactivity of the Mice IgG antibody anti-r-ZIKV-NS2B3. ELISA plates were sensitized with Column 1- r-ZIKV-NS2B (0.6 μg/well); Column 2- ZIKV from Vero cell culture (1:100), virus suspension: 0.1 M sodium carbonate buffer, pH 9.5, v/v; Column 3- Sera of patients with Zika fever (*n* = 28); Column 4: Sera from patients with Dengue virus were analyzed in duplicate. The assay was read at 450 nm. Graphical results represent the duplicate average of each sample and the cutoff was calculated by the average of absorbance of negative samples (*n* = 10) added 3 times the standard deviation of negative samples. The Tukey test was used as a post-test. Different letters represent statistical differences between groups (*p* < 0.05). Mice anti-r-ZIKV-NS2B3 IgG was used in this assay.

Various sera from the Dengue fever-positive group had as high an absorbance as many of the samples from the Zika fever-positive group. These data confirm the findings of Ravichandran et al.,^60^ who found low to moderate reactivity of ZIKV peptides in the sera of Dengue convalescents. As members of the Flaviviridae family, Zika and Dengue viruses are expected to share some degree of homology between their proteins and the proteins of other viruses in the same family (Figure 6).

According to Kikuti et al.,^67^ cross-reactions with other circulating arboviruses in tropical and subtropical regions have hindered the assembly of large subsets of Zika case samples to evaluate the precision of diagnostic tests. Commercial assays for ZIKV NS1 confirm these data, as an IgM capture assay (CLIA LIAISON XL Zika Capture IgM II, Diasorin, Italy) showed remarkable sensitivity (100%) for the diagnosis of Zika fever but cross-reacted with sera from patients recently infected with Dengue virus, Chikungunya virus, measles virus, and parvovirus B19.^68^

In this context, it is clear that the r-ZIKV-NS2B protein is capable of being recognized by antibodies against Dengue and Zika viruses. The cross-reactivity of antigens between Dengue and Zika viruses, including nonstructural proteins, is well-known.^69,70^ Various studies have reported that due to the relatedness between Dengue and Zika viruses (even sharing the same transmission vector, *Aedes aegypti*), it is not uncommon for structural molecules to cross-react between these two viruses.^70−72^ However, despite this cross-reactivity, it became evident that the r-ZIKV-NS2B protein has a significant ability to detect the presence of specific antigens present in Dengue or Zika viruses, identifying 100% of positive serum samples for both viruses. Conversely, all samples negative for both viruses were not detected. This result demonstrates the potential of the r-ZIKV-NS2B protein as a reliable molecule for determining the presence and absence of Dengue and Zika viruses.

**Figure 7:**
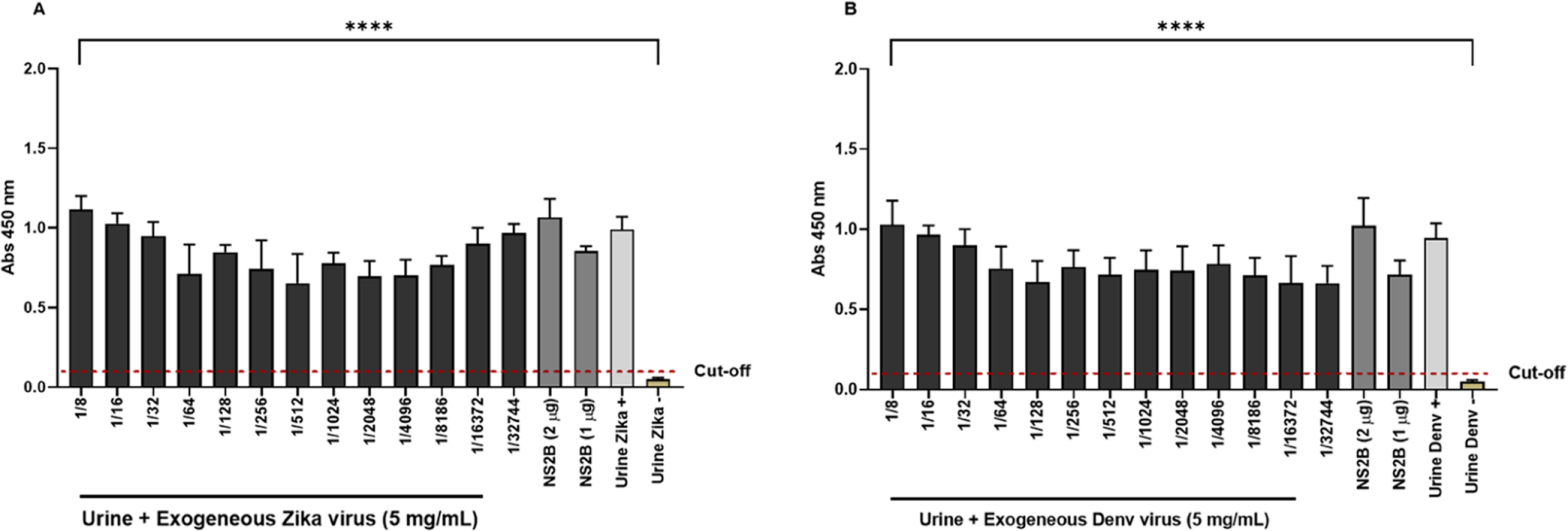
Detection of DENGV and ZIKV in urine samples. ELISA plates were sensitized with Column ZIKV (A) or DENGV (B) (500 μg/well), r-ZIKV-NS2B (1 or 2 μg/well). All samples were from were analyzed in triplicate. The assay was read at 450 nm. Graphical results represent the duplicate average of each sample and the cutoff was calculated by the average of absorbance of negative samples added 3 times the standard deviation of negative samples. Mice anti-r-ZIKV-NS2B3 IgG was used in this assay.

Continuing with the study on the use of the r-ZIKV-NS2B protein for the diagnosis of Dengue and Zika Virus, in addition to blood samples, the presence of viral antigens in urine samples from positive and healthy patients was evaluated. The results obtained revealed that the antibodies produced by the r-ZIKV-NS2B protein were effective in detecting specific viral antigens present in the urine of patients who were positive for both viruses. In contrast, in samples from patients negative for both viruses, there was no detection of antigens (Figure 7).

The specificity of polyclonal antibodies and possible interference of the urine sample in the immunological assay was evaluated using wells sensitized with 1 and 2 μg of purified r-ZIKV-NS2B. As a result, it was shown that r-ZIKV-NS2B diluted in the urine of patients negative for Zika and Dengue was detected by polyclonal antibodies. Thus, it is possible to observe that the reactions detected in the ELISA assay were actually obtained by reactions between the polyclonal antibodies produced and the antigens present in the urine samples (used to sensitize the plaques), not being the result of a possible nonspecific interaction between antibodies and nontarget molecules present in patients’ urine (Figure 7).

To titrate the antibodies produced, an aliquot of urine from a healthy patient is added to a viral aliquot of Dengue or Zika and is used to sensitize the wells. After the plates were read, it was seen that the target protein was capable of being detected by polyclonal antibodies with dilutions ranging from 1/8 to 1/32,744, with a statistical difference for the negative control group. This result shows that polyclonal antibodies show great reactivity toward the antigens present in Dengue and Zika viruses, even at low concentrations (Figure 7).

Currently, the gold standard test for virus detection is the PCR test. The detection of Dengue and Zika viruses also fits this context. As an alternative to the PCR test, which requires specialized structure and labor, diagnostic tests have been widely used due to their speed and safety, such as commercial diagnostic kits used in the COVID-19 pandemic.^73−77^

Regarding the use of diagnostic tests that use the detection of antibodies or viral antigens, it is still necessary to observe the type of sample used. Among the possibilities, we have samples with more invasive collections, such as blood samples. But, taking into account less invasive collections, we have growing research that seeks to use saliva and urine as samples to be used to detect viral diseases, including Dengue and Zika virus.^27,78−81^

In addition to the great structural similarity, the Dengue and Zika viruses generate infections in humans that can present similar symptoms.^82,83^ Although similar, once the infections are established, medical treatment for both viral infections cannot be exactly the same. For example, patients with Dengue fever have an increased risk of bleeding, an event known as “Dengue hemorrhagic fever”.^84−86^ In this context, the use of acetylsalicylic acid (which belongs to the group of nonsteroidal anti-inflammatory drugs with analgesic, antipyretic, and anti-inflammatory properties) is contraindicated as it inhibits the cyclooxygenase enzyme, reducing the production of thromboxane A2, an aggregation stimulator platelets (increasing the risk of bleeding).^87−90^

## 3 Conclusions

**Figure 8:**
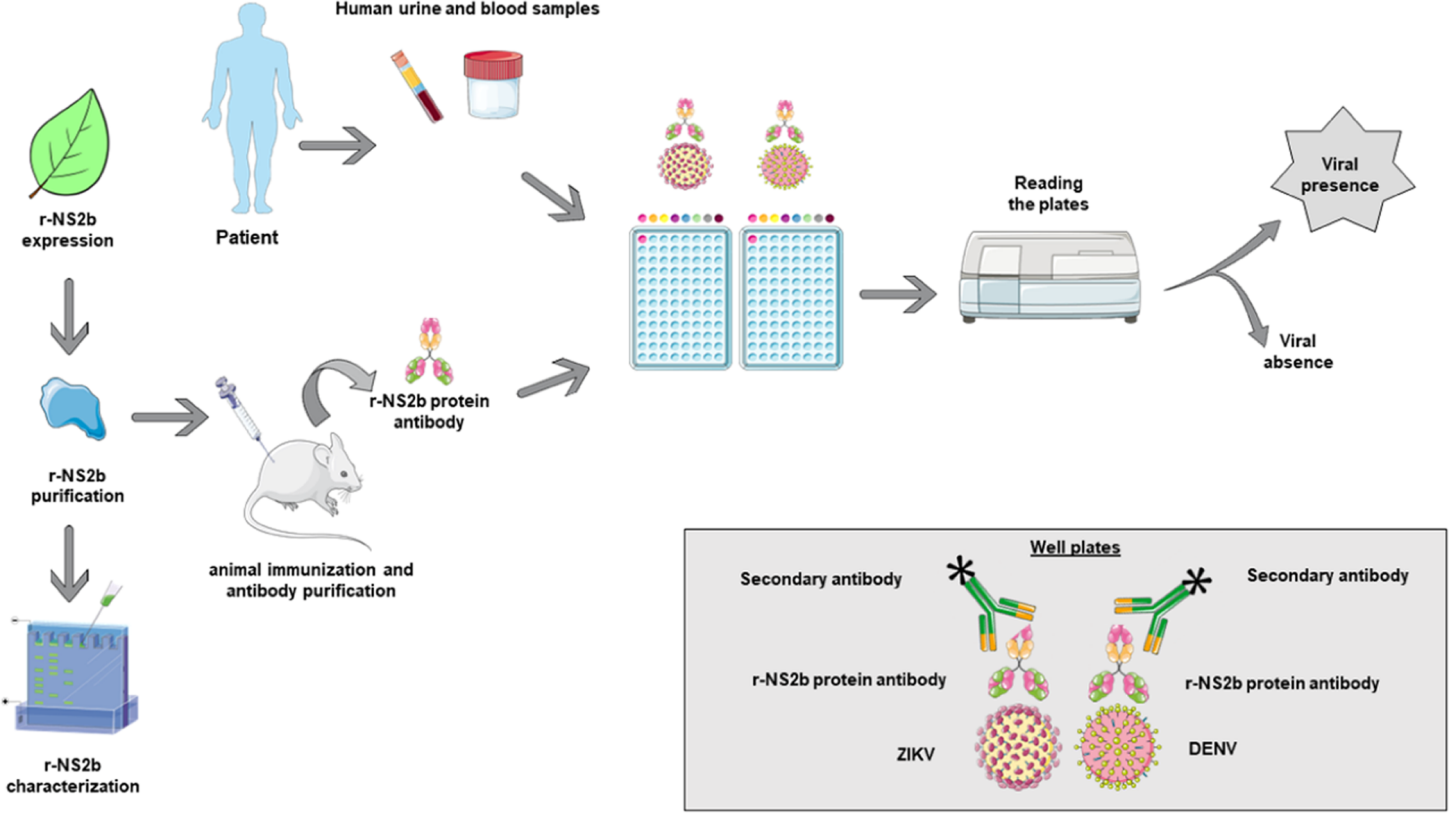
Schematic flow of recombinant protein expression, antibody production, and detection. This figure were drawn using images from Servier Medical Art. Servier Medical Art by Servieris licensed under a Creative Commons Attribution 4.0 Unported License (CC BY) (https://smart.servier.com/citation-sharing/) (https://creativecommons.org/licenses/by/4.0/).

In conclusion, based on the results obtained in this research, it was demonstrated that polyclonal antibodies (IgG) arising from immunization with the recombinant NS2B protein of Zika virus expressed in *N. benthamiana* has the potential to recognize viral infections of arboviruses caused by Dengue and Zika virus. Such detection proved to be efficient in blood and urine samples, making it possible to clearly identify patients with the presence or absence of arboviruses (Figure 8).

## 4 Material and Methods

### 4.1 Medium Preparation

The composition of the YM medium consisted of 0.04% Yeast extract, 1.0% mannitol, 1.7 mM NaCl, 0.8 mM MgSO_4_, and 2.2 mM K_2_HPO_4_. For the infiltration medium, YM medium was supplemented with MES buffer, pH 5.6 (Sigma), and acetosyringone (Sigma) to achieve final concentrations of 10 mM and 100 μM, respectively. The Gamborg’s solution was comprised of 10 mM MES, 200 μM acetosyringone, 20 g/L sucrose, and 3.2 g/L Gamborg’s B-5 Basal Medium (Sigma).^91^

### 4.2 Plant Material

*Nicotiana benthamiana* plants were grown in a climate-controlled room (26 °C) with a light-dark cycle of 16 h/8 h and relative humidity of 40–50%. The seedlings were grown for about 6–8 weeks before agroinfiltration.

### 4.3 Bacterial Strains

*Escherichia coli* DH10B was cultured in Luria–Bertani LB (Sigma) agar containing the selective antibiotic ampicillin (Sigma) at 37 °C for 24 h. *Agrobacterium tumefaciens* strain LBA4404 (Invitrogen) was cultured on YM agar media containing kanamycin, streptomycin, or rifampicin (25, 50 μg/mL or respectively). The plates were incubated for 3 days at 28 °C.

### 4.4 Zika Virus and Dengue Virus Isolation

ZIKV and DENV were obtained from LACEN/CE (Central Public Health Laboratory of Ceará, Brazil) of patients with Zika or Dengue PCR-positive. Next, all viruses were replicated in Vero cells, as described below. Initially, 90–100% confluent Vero cell culture in 25 cm^2^ tissue culture flasks was infected for 2 h with ZIKV or DENV. After infection, the cells were maintained at 37 °C in an L-15 medium (Cultilab, Brazil) containing 2% fetal bovine serum (Cultilab, Brazil) and 1% penicillin/streptomycin (Sigma). After 7 days of incubation, the medium containing the virus was removed and stored at −80 °C. The replication of the virus was verified by RT-PCR of the sample before and after replication.

### 4.5 Sera Samples

All sera samples were provided by the LACEN/CE (Central Public Health Laboratory of Ceará, Brazil) and confirmed ZIKV or DENV positive, at least by RT-PCR. Sera samples were divided into the following groups: (ZIKV+) Sera of positive Zika fever patients (*n* = 20); (DENV+) Sera of positive Dengue fever patients (*n* = 25); and (C−) Sera of ZIKV and DENV PRC negative (*n* = 13).

### 4.6 Urine Samples

All urine samples were provided by the LACEN/CE (Central Public Health Laboratory of Ceará, Brazil) for patients confirmed to be ZIKV or DENV positive at least by RT-PCR. Urine samples were divided into the following groups: (ZIKV+) Urine of positive Zika fever patients (*n* = 3); (DENV+) Urine of positive Dengue fever patients (*n* = 3); and (C−) Urine of ZIKV and DENV PRC negative (*n* = 3).

### 4.7 NS2B Sequence and Synthesis

The sequence of the NS2B protein gene of Zika virus was obtained from the National Center for Biotechnology Information (NCBI) (accession number KU497555). It was then optimized for expression in *N. benthamiana* and chemically synthesized (Biobasic Inc., Canada). The sequence was flanked by the AttL1 and AttL2 sites so that it could be cloned into the plant pCAMGate-ER-HFBI binary expression vector using Gateway LR Clonase Enzyme Mix II technology (Invitrogen, 11791020).^92^ The sequence synthesized by Bio Basic Gene Synthesis was provided in delivery plasmid pUC57 (GenBank: Y14837.1) with antibiotic resistance to ampicillin.

### 4.8 Expression System

The recombination product was transformed into chemically competent *E. coli* DH10B. They were grown at 37 °C in LB plates used for plasmid DNA amplification, and a positive clone was selected by colony PCR. The plasmid pCAMGate-ER-NS2B-HFBI was transformed into chemically competent *A. tumefaciens* LBA4404 (Invitrogen, 18313015) and then grown in YM medium (0.04% Yeast Extract, 1.0% mannitol, 1.7 mM NaCl, 0.8 mM MgSO_4_, and 2.2 mM K_2_HPO_4_) with pH adjusted to 7.0 and selective antibiotics. Upon recombination, the 3′ end of the subcloned ZKV NS2B gene was fused to the HFBI gene by a ligand 3 (GSSS). Expression, the construct also exhibits a human C-myc detection/purification tag and an endoplasmic reticulum-targeting tetrapeptide KDEL signal peptide and a nopaline synthase transcription terminator at its C-terminus^92^ (Figure 1).

### 4.9 Agroinfiltration

A single *A. tumefaciens* colony from a fresh dish was harvested to produce an inoculum containing the vector carrying the DNA of interest, the “positive transformant”. The same was done with an *A. tumefaciens* clone containing the coding sequence for the silencing inhibitor Cymbidium virus ringSpot p19 (CymRSV).^44^ The cultures were performed into 2 mL of YM broth containing kanamycin and streptomycin or rifampicin selection antibiotics, respectively, and grown overnight at 28 °C in a shaker incubator. Then, the starter culture was used in 50 mL of infiltration medium (YM), supplemented with MES buffer (pH 5.6) (Sigma, M3671) and acetosyringone (Sigma, D134406) at a final concentration of 10 mM and 100 μM, respectively. Then, the bacterial cultures were incubated overnight at 28 °C (240 rpm). After reaching an optical density at 600 nm (OD_600_) of 0.8–1.0, the cultures were centrifuged (2500*g*, 15 min, 24 °C) and the cell pellets were resuspended in Gamborg’s solution (10 mM MES, 200 μM acetosyringone, 20 g/L sucrose, and 3.2 g/L Gamborg basal medium B-5 (Sigma G5893))^91^ to an OD_600_ = 0.6. After 1 h of incubation with gentle shaking at room temperature, the resuspended cells were mixed with the *Agrobacterium* p19 suspension (1:1, v/v). For plant infiltration, leaves of 6–8 weeks old from *N. benthamiana* plants were infiltrated by applying pressure to the abaxial surface of the leaf with a 1 mL disposable syringe containing the *Agrobacterium* suspension. The agroinfiltrated plants were incubated for 4–7 days after infiltration (DAI). Then, the agroinfiltrated leaves were harvested individually and stored at −80 °C until use. Control plants were infiltrated with the p19 culture only. Plants were maintained at 25 °C with a 16 h photoperiod and in a hydroponic solution throughout the experiment.

### 4.10 r-ZIKV-NS2B Extraction

For protein extraction, the leaf tissue was macerated in a crucible mortar with a pestle in the presence of liquid N_2_ until very fine flour was obtained. The macerate was placed in cold extraction buffer [PBS containing 0.1% Triton X-100 (Sigma, T8787) and 10 mM PMSF (Sigma, P7626)] at a ratio of 1:6 (w/v) for 20 min at 4 °C. Then, the mixture was centrifuged at 5000*g* for 15 min at 4 °C. The precipitate was discarded, and the supernatant (total extract) was stored at −20 °C for further analysis.^35^

### 4.11 r-ZIKV-NS2B Purification

An Aqueous Two-Phase System (ATPS) was optimized for semipurification of the r-ZIKV-NS2B protein to determine the best condition for protein separation by hydrophobic characteristics. Triton X-114 (Sigma, X-114) was added to the extract to the final detergent concentrations at 2, 4, 6, or 8% (v/v), and the mixtures were vortexed and then incubated at 30 °C until the phase separation was stable (approximately 30 min or until the lower phase of Triton X-114 showed a 10-fold increase in initial surfactant volume). The upper aqueous phases were stored for later analysis, while to the lower phase was added isobutanol (Sigma, 33064) at a 1:1 (v/v) ratio. After homogenization, the mixture was centrifuged (3200*g*, 15 min, 24 °C), the upper (alcohol phase) and the insoluble middle phase were discarded, and the (lower) aqueous phase containing semipurified r-ZIKV-NS2B was set aside for further analysis.^99−101^

The ATPS fraction (2.5 mg in 2 mL of equilibrium buffer) was loaded onto the HiTrap Phenyl HP column (GE Healthcare), connected to the kta Start System (GE Healthcare), and pre-equilibrated with 0.02 M sodium phosphate buffer, pH 7.2, containing 1.0 M (NH_4_)_2_SO_4_ (Flow: 1.0 mL/min, Pressure: 0.3 MPa, Fraction: 1.5 mL). The nonretained proteins were eluted with the equilibrium buffer, and the adsorbed proteins were eluted by decreasing the concentration gradient of (NH_4_)_2_SO_4_.^35^

### 4.12 Polyacrylamide Gel Electrophoresis and Protein Quantification

The electrophoretic profile of crude extract and the purified protein was observed in polyacrylamide gel in the presence of SDS (SDS-PAGE), according to the methodology described by Laemmli^93^ adapted to the use of plates. For the assembly of the plates, application gel was used, enclosing 4% acrylamide and 1% SDS prepared in 0.5 M Tris-HCl buffer, pH 6.8, and separation gel containing 12 or 15% acrylamide and 1% SDS in 3.0 M Tris-HCl buffer, pH 8.8. The samples were first dissolved in sample buffer (0.0625 M Tris-HCl, pH 6.8, containing 1% SDS, 20% glycerol, and bromophenol blue) and added 2% β-mercaptoethanol. They were then heated at 98 °C for 2 min and centrifuged at 10,000*g* for 5 min at room temperature. Then, aliquots of each sample were applied to wells, and the run was conducted at a constant voltage of 180 V and a current of 400 mA for approximately 1 h. The protein bands were stained with Coomassie blue solution (0.25% Coomassie brilliant blue; 45% methanol; 10% acetic acid) for 1 h and bleached with bleaching solution (30% methanol and 10% acetic acid). Molecular mass standards phosphorylase B (97 kDa), bovine serum albumin (66 kDa), ovalbumin (45 kDa), carbonic anhydrase (30 kDa), soybean trypsin inhibitor (20.1 kDa), and 2-lactoalbumin (14.4 kDa) were added to the extract. Protein samples were quantified in the gel by Quantity One 29.0 software (BioRad), using different amounts of the BSA protein (0.25 μg, 100 μg, and 50 μg) as a pattern. The quantification was used for further analysis.

### 4.13 Soluble Protein Quantification

The soluble proteins were quantified based on a standard curve of bovine serum albumin, following the methodology described by Bradford.^94^

### 4.14 Animal Immunization

In this experiment, 6 mice (female Balb/c, age 6–8 weeks, 25–30 g) obtained from Nucleus of Experimental Biology (Nubex) of the University of Fortaleza were used after a week of acclimatization. The mice were maintained at room temperature (25 ± 1 °C) and a light-dark cycle of 12 h. After acclimatization, to produce anti-r-ZIKV-NS2B polyclonal antibodies, the immunization of mice was carried out using 30 μg of the purified r-ZIKV-NS2B with complete Freund’s adjuvant (Sigma) (1:1) for subcutaneous (sc) injection. Trial bleeding occurred on day 0, with a boost of sc injection with incomplete FA at day 21, and another trial was bleeding on day 26. After the antibody titration, another boost with intraperitoneal (IP) injection was 35 with the antigen only. To obtain the antisera, the animals had their blood collected by the retro-orbital plexus on day 0 (preimmune) and the 42 day after the start of immunization.^95^

### 4.15 Purification of IgG Antibodies

After sample collection, microtubes (without anticoagulants) containing the blood were left at rest in a vertical position for 4 h at room temperature. Following erythrocyte coagulation, the supernatant was carefully collected and transferred to new microtubes. Subsequently, the samples were centrifugated (3000*g*, 10 min, 4 °C), with subsequent collection of the supernatant. The purification of IgG antibodies occurred through affinity chromatography on a Protein G matrix (HiTrap, MERCK) coupled to a KTA Start system (GE). Purification procedures followed the manufacturer’s manual instructions.

### 4.16 Western Blotting

Proteins were first subjected to SDS-PAGE separation (15%) as previously described and then transferred to a nitrocellulose membrane (Amersham Protran, GE Healthcare) in a transfer buffer semidry electrotransference vessel [39 mM glycine, 0.0375% (w/v) SDS and 20% (v/v) methanol in 48 mM Tris-HCl buffer (pH 8.0)], under constant amperage of 300 mA for 1 h. After transfer, nonspecific neutralized sites present in the membrane were blocked using a blocking solution [PBS containing 5% skim milk (w/v)] overnight. Following blockage, the membrane was incubated with the primary anti-c-Myc monoclonal antibody (Genscript, A00864, Piscataway) produced in mice at a dilution of 1:2,500 (v/v) for 1 h at room temperature. Subsequently, the membrane was rinsed 3 × 10 min in PBS-T [0.05% Tween 20 (Sigma, P9416) in PBS] under continuous agitation to remove the unbound primary antibody.

Next, the membrane was then incubated with peroxidase-conjugated secondary antibody (peroxidase-conjugated anti-mouse IgG) (Invitrogen, G21040) at a dilution of 1:5000 (v/v) in PBS-T for 1 h at 4 °C with slow agitation. To remove unbound secondary antibody, the membrane was again washed with PBS-T as described above. For development, the membranes were immersed in Clarity Western ECL Substrate (BioRad) kit reagents at a 1:1 (v/v) ratio. Alternatively, membrane development was performed with DAB (3,3′-diaminobenzidine tetrahydrochloride). To verify the integrity and effect of HFB peptide fusions, Western blotting was performed using the Zika virus anti-NS2b polyclonal antibody (GENETEX) as well as human serum from ZIKAV-positive patients.

### 4.17 Enzyme-Linked Immunosorbent Assay (ELISA) for r-ZIKV-NS2B with Human Sera

The recombinant protein was diluted into a coating buffer (0.1 M sodium carbonate buffer, pH 9.5) to the concentration of 10 ng/μL. Then, 100 μL of the diluted extract was used to coat each well of the microplates (Sigma, M9410). After overnight coating, the plates were washed with PBS-T, blocked for 1 h with 1% gelatin (Sigma, G6650), and then washed three times with PBS-T. To recognize the antigen, it was used sera from 20 IgM ZIKV-positive patients obtained from the Central Laboratory of Public Health in the state of Ceara (LACEN/CE, Brazil) and 25 sera IgM DENG positives MAC-ELISA IgM (Panbio, Australia). As appositive control was used, ZIKV from cell cultures and as negative control was used sera from 25 children 2–4 years without contact with the disease. All sera were used at a 1:100 (v/v) dilution and tested in duplicates. After incubating the sera for 1 h at 37 °C, another washing step was performed, and 100 μL of peroxidase-conjugated anti-IgM (Sigma) (1:5000) was added. After a final washing step, 100 μL of TMB solution (Thermo Fisher, 34028) was added to each well, and the plates were incubated for 20 min in the dark. The absorbance at 650 nm was read using a microplate reader (Synergy 2, Biotek).^96^ After calculating the average value of 650 nm absorbance of the negative samples and its standard deviation, the test’s cutoff was established as the average absorbance of these plus 3 times its standard deviation.^97^

The test’s cutoff was defined as the average absorbance of the negative samples plus 3 times its standard deviation.^97^ Samples with absorbance equal to or below the cutoff value were qualified as negative ones, and then, the sensitivity and the specificity of the test were determined.^98^ Briefly, all 37 sera were divided into four groups according to their results on the NS2B-ER-HFBI ELISA test: true positives (TP), false negatives (FN), false positives (FP), and true negatives (TN). Then, the sensitivity [sensitivity = TP/(TP + FN)] and specificity [specificity = TN/(TN + FP)] of the test were calculated.

### 4.18 Enzyme-Linked Immunosorbent Assay (ELISA) for r-ZIKV-NS2B with Human Urine

#### 4.18.1 Urine Sample Preparation

In order to ensure the removal of potential impurities contained in the urine samples, the samples underwent the following processing steps. Initially, 1 mL of urine was placed in a plastic microtube (2 mL) and centrifuged for 10 min at 10,000*g*, 4 °C. Following centrifugation, the supernatant was collected and transferred to another microtube (2 mL). Subsequently, the urine sample was subjected to a second centrifugation (10,000*g*, 10 min, 4 °C). Finally, the supernatant was once again collected and used for the immunoassays.

#### 4.18.2 Enzyme-Linked Immunosorbent Assay (ELISA)

In this assay, 2 or 1 μg of recombinant NS2B was diluted in 100 μL of carbonate buffer (0.1 M sodium carbonate buffer, pH 9.5). Simultaneously, a solution containing urine from a healthy patient and Zika virus (supernatant of virus cell culture = 5 mg/mL), in a 1:1 (v/v) ratio, was equally mixed (1:1, v/v) with carbonate buffer (0.1 M sodium carbonate buffer, pH 9.5). After the prior preparation of these solutions, 100 μL of urine (with serial dilution of 1/8-1/32744) or NS2B (2–1 μg) were applied to 96-well plates (Sigma, M9410) for sensitization. The plates were left to rest for 12 h at 4 °C. Then, the samples were discarded, and the wells were washed 3 times with PBS buffer with 0.05% Tween (Sigma). After washing, the plates were incubated with 100 μL of primary antibody (IgG anti-NS2B) produced in mice (1/1000, v/v) diluted in PBS for 2 h at 28 °C with gentle agitation. After they were incubated with the primary antibody, the wells were washed with PBS-T three times. Subsequently, an aliquot of 100 μL of secondary antibody anti-IgG coupled to peroxidase (1/1000, v/v, diluted in PBS) was dispensed into each well, remaining in contact for 2 h at 28 °C with gentle agitation. After this period, the wells were washed again with PBS-T. Finally, 200 μL of TMB (Thermo Fisher) was applied to each well. Plate readings were taken after 15 min using a microplate reader (Synergy 2, Biotek) at a wavelength of 450 nm. Urine from Zika-positive patients (*n* = 3) and Zika-negative patients (*n* = 3) were used as positive and negative controls, respectively. Assays with urine from Dengue-positive patients were carried out following exactly the procedures described above. Urine from Dengue-positive patients (*n* = 3) and Dengue-negative patients (*n* = 3) were used as positive and negative controls, respectively.

### 4.19 Statistical Analysis

All analyses were performed using Prism version 8.0 (GraphPad Software, Inc., La Jolla, CA). One-way ANOVA by Tukey’s Multiple Comparison Test was used to analyze means with statistical differences. All *p*-values <0.05 were considered as statistically significant. The data is presented as mean ± standard deviation (SD). The experiments were conducted in triplicates.

## Notes

The Institutional Research Ethics Committee (CEP), State University of Ceara-Brazil, approved all experiments (Approval 04510970/2019), which were carried out in accordance with the Declaration of Helsinki.
